# Reliable UHF Long-Range Textile-Integrated RFID Tag Based on a Compact Flexible Antenna Filament

**DOI:** 10.3390/s20123435

**Published:** 2020-06-17

**Authors:** Mahmoud Wagih, Yang Wei, Abiodun Komolafe, Russel Torah, Steve Beeby

**Affiliations:** 1 School of Electronics and Computer Science, University of Southampton, Southampton SO17 1BJ, UK; A.O.Komolafe@soton.ac.uk (A.K.); rnt@ecs.soton.ac.uk (R.T.); spb@ecs.soton.ac.uk (S.B.); 2 School of Science and Technology, Nottingham Trent University, Nottingham NG11 8NS, UK; yang.wei@ntu.ac.uk

**Keywords:** antenna, electrically-small antennas, E-textiles, impedance matching, Internet of Things, RFID, textile antenna

## Abstract

This paper details the design, fabrication and testing of flexible textile-concealed Radio Frequency Identification (RFID) tags for wearable applications in a smart city/smart building environment. The proposed tag designs aim to reduce the overall footprint, enabling textile integration whilst maintaining the read range. The proposed RFID filament is less than 3.5 mm in width and 100 mm in length. The tag is based on an electrically small (0.0033$\lambda^{2}$) high-impedance planar dipole antenna with a tuning loop, maintaining a reflection coefficient less than −21 dB at 915 MHz, when matched to a commercial RFID chip mounted alongside the antenna. The antenna strip and the RFID chip are then encapsulated and integrated in a standard woven textile for wearable applications. The flexible antenna filament demonstrates a 1.8 dBi gain which shows a close agreement with the analytically calculated and numerically simulated gains. The range of the fabricated tags has been measured and a maximum read range of 8.2 m was recorded at 868 MHz Moreover, the tag’s maximum calculated range at 915 MHz is 18 m, which is much longer than the commercially available laundry tags of larger length and width, such as Invengo RFID tags. The reliability of the proposed RFID tags has been investigated using a series of tests replicating textile-based use case scenarios which demonstrates its suitability for practical deployment. Washing tests have shown that the textile-integrated encapsulated tags can be read after over 32 washing cycles, and that multiple tags can be read simultaneously while being washed.

## 1. Introduction

Integrated electronic functionality in textiles, known as e-textiles, is seen as an emerging enabling platform for wearable sensing, computing and wireless communication [1]. A key requirement of e-textiles is durability, seamless integration in the user’s garment and compatibility with standard textile manufacturing [2]. To enable such e-textiles to coalesce in the Internet of Things (IoT) and Internet of Everything (IoE) paradigms, reliable Radio Frequency (RF) front-ends need to be developed to meet the requirements of wearable textile-based mobile computing and enable reliable wireless links to e-textiles.

Radio Frequency Identification (RFID) has been widely adopted as a communication and identification standard for low-cost distributed IoT systems [3,4,5,6,7]. UHF RFID, utilizing impedance-matched radiative antennas and passive RFID integrated circuits (ICs) enables read-range ranges in excess of few meters [3]. Various passive sensing [8] and power-autonomous RF-powered sensors [9,10] have been developed based on off-the-shelf RFID readers to enable battery-free smart devices.

RF e-textiles and wearable antennas have been widely researched for various applications [11]. Textile and flexible antennas for Ultra-High Frequency (UHF) RF Identification (RFID) [12,13,14,15,16,17], 2.4 GHz Body Area Networks (BAN) antennas based on dispenser and inkjet printing [18,19,20] as well as photolithography [21], in addition to 5G millimeter-wave applications from 24 GHz up to 60 GHz [22,23,24] have been previously presented. Furthermore, such Electromagnetic (EM) systems operating in vicinity of the body need to be compliant with the Specific Absorption Rate (SAR) regulations [25]. Nevertheless, most reported textile and flexible antennas have not been integrated in the textile, where they are normally printed or adhered to the garment’s surface. Most reported textile microstrip antennas occupy large area (approximately half the wavelength in length and width) and require substrates thicker than 1 mm to achieve a high radiation efficiency [20]. While fully-knitted textile RFID antennas have been presented, their resilience to washing and bending has yet to be investigated [26]. In addition, knitting may affect the mechanical robustness of the interconnects between the RFID IC and the antenna.

The design of a RFID antenna is limited by multiple parameters. First of all, the impedance of the antenna needs to be matched to the transponder IC to achieve a low power reflection coefficient. Moreover, the antenna’s gain needs to maximized by improving the total efficiency and through adjusting the radiation patterns to the proposed application [3]. When it comes to implementing the RFID antenna on flexible and wearable materials, additional limitations are imposed on the size of the antenna which may limit the performance. For example, while a 3D-printed low-cost RFID antenna has been proposed in Reference [27] with a 26 m read range, the antenna occupies a large area of 140×60 mm${}^{2}$ and is not planar, prohibiting its integration in pervasive wearable and retail applications.

A novel e-textiles fabrication method for integrating electronics in textiles using a standard textile weaving process and a flexible circuit manufacturing process has been proposed in Reference [2]. This approach relies on the flexible circuits being long, thin filaments that can be subsequently woven into a textile. The width of the filament must be minimized in order to achieve invisible integration of the circuit within the textile and this geometric requirement places constraints on the design of the antenna. In Reference [28], a coplanar waveguide (CPW) antenna has been presented using the glob-top packaging approach detailed in Reference [2] showing no degradation in the antenna’s impedance matching after concealment in the textile. However, the mechanical robustness of this was not evaluated and the glob top approach has previously been found to provide inadequate protection during mechanical loading and machine washing [29].

This work presents the first implementation of a thin and flexible RFID antenna designed for invisible integration within a woven textile and encapsulated using a proven technique for achieving reliability and washability [29]. An ultra-compact flexible RFID tag is presented and integrated in a standard woven textile to realize a fully concealed, washable and reliable RFID strip. The proposed antenna is the smallest RFID dipole antenna and represents the first implementation of a functional RFID antenna that is fully concealed in the textile. The tag achieves a read range of 8.2 m, which is three times improvement over the state of the art when normalized to the antenna’s area. In addition to washing, the textile-integrated tag has been subjected to harsh reliability tests to demonstrate its suitability for use in real world applications. The proposed fabrication method enables a reliable assembly based on standard electronics manufacturing processes as well as an integrated textile solution using textile manufacturing methods.

## 2. RFID Tag Design

### 2.1. Chip Selection

To realize a flexible RFID tag which can be concealed in textiles, a RFID chip needs to have a small footprint and a low-profile, and have a minimum number of pads that have a large clearance making them easy to attach. This allows for manufacturing tolerances and ensures the contact resistance between the RF pads and the antenna can be minimized. The NXP UCODE 7 was selected based on the above criteria. The IC has an area of 0.5 × 0.5 mm${}^{2}$ and a 358 μm clearance between the pads. The selected IC is designed for the 915 MHz Industrial, Scientific and Medical (ISM) band with an input impedance of $Z_{IC}=11.8-j254\Omega$.

### 2.2. Antenna Design

The performance of a RFID tag is described by the read range, calculated using (1) as a function of the antenna’s reflection coefficient $S_{11}$, tag’s gain $G_{r}$, transmitted Equivalent Isotropic Radiated Power (EIRP) combining the transmitter (the reader) antenna gain and transmitted power $G_{t}P_{t}$, in addition to the power threshold $P_{\mathrm{th}}$, also known as the read/write sensitivity of the particular RFID chip. For the NXP UCODE 7, this is −21 and −16 dBm for read and write respectively [30].

(1) $$r=\frac{\lambda }{4\pi }\sqrt{\frac{G_{r}G_{t}P_{t}}{P_{th}}\times (1-|S_{11}{|}^{2})}.$$

From (1), an impedance match between the antenna and the RFID chip is crucial to achieve a sufficiently-low power reflection coefficient |S${}_{11}$|${}^{2}$. This is achieved by designing an antenna to directly conjugate the chip’s impedance where $Z_{ant}=Z_{IC}^{*}$. Complex-impedance inductive antennas have been previously used for RFID [4] as well as RF energy harvesting applications [31] where a highly capacitive device needs to be matched. Furthermore, the antenna’s dimensions should be comparable to the wavelength, to maintain a higher receiver gain $G_{t}$ and maximize its aperture efficiency.

**Figure 1:**
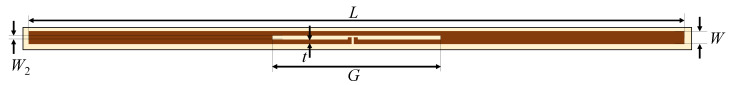
Layout of the proposed Radio Frequency Identification (RFID) antenna. Dimensions in mm: L=109.0, W=3.5, $W_{2}=2.0$, G=28.0, t=0.75.

A dipole antenna with an inductive matching loop can be designed to achieve scalable inductive impedance, which can be designed to conjugate the impedance of the RFID IC [4]. A long dipole (approaching λ/2) combined with a T-matching inductive loop can achieve a fully-inductive impedance across a broad range of frequencies [4]. Using the built-in inductive loop geometry of the folded dipole, the antenna’s impedance can be tuned to closely match the RFID IC at 915 MHz. In addition, as the target fabrication process is more optimized for single-sided circuits, a dipole or a coplanar antenna is preferred for ease of waving in textiles. The proposed antenna design is based on a single-sided planar dipole antenna shown in Figure 1. The dimensions of the antenna, such as the length and the width of the conductive traces and the gap have been optimized numerically, as presented in Section 4, to achieve an input impedance close to the complex conjugate of the IC’s impedance. While infinitesimally-thin wire dipole antennas could be designed to achieve a close input impedance match and high radiation efficiency, they would not be compatible with the proposed fabrication technique for reliable integration in e-textiles. To explain, fabricating thin wire antennas with a high resolution is a complex process compared to 2D photolithography of commercially-available laminates. In addition, thin wires will not be resilient to multiple bending cycles or washing making them unsuitable for wearable and e-textile applications.

## 3. Tag Fabrication and Encapsulation

### 3.1. Tag Fabrication

The antenna has been fabricated using a novel fabrication process utilizing standard photolithography to realize ultra-thin and narrow single-sided flexible circuit filaments. The flexible circuit can then be encapsulated and concealed in a standard textile weave using textile-manufacturing- compatible methods. Commercial copper-clad polyimide (Kapton) laminates of a thickness o 25 μm, with 16 μm epoxy adhesive and 18 μm copper are utilized. In order to provide compatibility standard photolithography equipment, a 6 inch silicon (Si) wafer is used as a carrier to support the copper-clad Kapton during the manufacturing process. Therefore, the copper-coated Kapton is cut into 150 mm diameter discs to match the size of the Si wafer. The main steps of the fabrication process are:

- A standard positive photoresist, S-1813, is spin-coated onto the Si wafers as an adhesive followed by attaching the copper coated Kapton disc onto the Si carrier.
- Photoresist is then spin-coated onto the copper layer and cured at 110 ${}^{\circ }$C for 3 min.
- The photoresist is UV exposed through the photomask for 30 s
- The UV-decomposed photoresist is developed using a 1:4 solution of AZ 400K developer and DI water.
- The copper is etched for 9 min using a standard PCB bubble etch tank.
- The remaining resist is removed and the exposed copper cleaned using acetone.
- The bare RFID IC is mounted using a standard soldering process.

**Figure 2:**
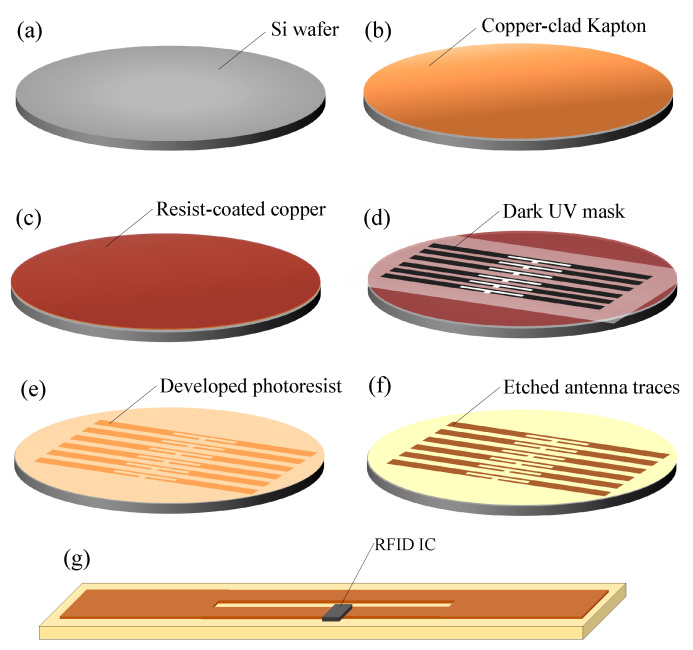
Fabrication steps of the Kapton RFID filament for textile-integration: (**a**) planar silicon wafer used as a carrier, (**b**) adhere copper-clad Kapton laminates to the Si wafer, (**c**) spin-coat photoresist, (**d**) UV exposure using a dark mask, (**e**) develop photoresist showing the traces pattern, (**f**) expose antenna traces after wet etching, (**g**) RFID filament tag with the mounted RFID IC.

**Figure 3:**
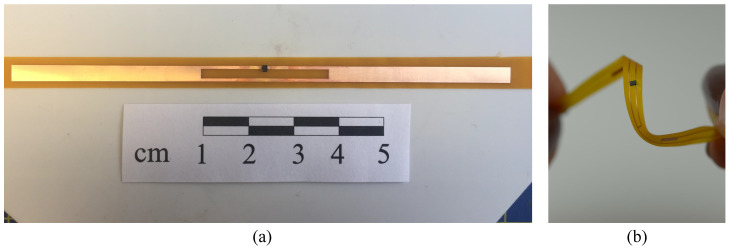
Photograph of the fabricated RFID prototypes: (**a**) the proposed RFID antenna with the mounted IC, (**b**) bending of an RFID tag fabricated using the proposed technique.

Figure 2 shows the fabrication steps of the flexible RFID tag filament. Photographs of the fabricated RFID tag and an RFID antenna, fabricated using the proposed technique, being bent are shown in Figure 3.

While the technique described above is a standard approach for the fabrication of Polyimide-based RF circuits [21,22,32], the molded encapsulation process, [29], and woven integration of the flexible antenna filament presents a unique approach that hides the tag in the textile and provides excellent reliability in practical applications.

### 3.2. Tag Encapsulation

The fabricated RFID filament is encapsulated using a thermally molded 50 μm-thick Kapton layer containing a cavity that matches the size and location of the RFID chip. The cavity is formed in the Kapton filament is enabled by a custom built steel mold containing male (top) and female (bottom) mold which are designed according to the geometry of the RFID IC. The Kapton is molded prior to assembling due to the high temperature involved that would otherwise damage the solder connections to the RFID IC. The procedure is as follows:

1. Insert 50 μm-thick Kapton filament into the trench in the bottom tool piece.
2. Locate the top tool piece thereby sandwiching the Kapton filament.
3. Place the sandwich into an oven at 360 ${}^{\circ }$C for 60 s to soften the Kapton.
4. Clamp the bottom and top inserts together to deform the Kapton into the shape of the RFID IC.
5. Allow to cool before detaching the Kapton.

**Figure 4:**
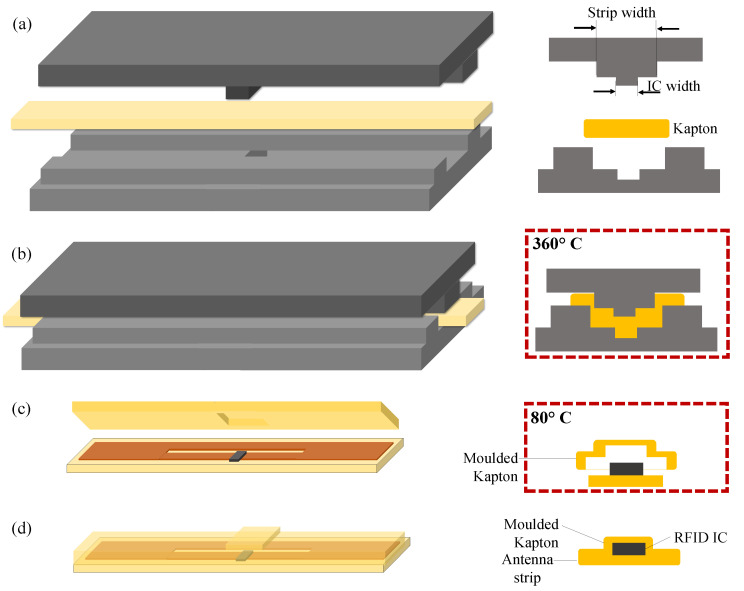
The proposed encapsulation technique of the RFID tag: (**a**) The bespoke Kapton molding tool, (**b**) molding the Kapton at 360 ${}^{\circ }$C, (**c**) the molded Kapton sandwich fitting onto the RFID filament, (**d**) the encapsulated RFID strip.

**Figure 5:**
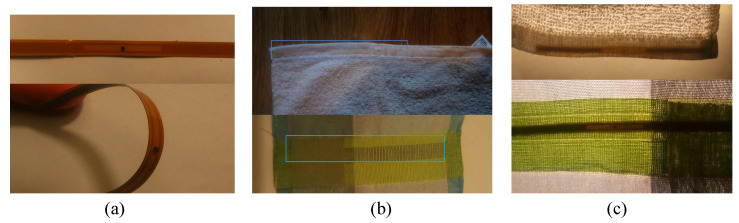
Photographs of the encapsulated RFID tags and the tags integration in textiles: (**a**) the Kapton-molded tag, (**b**) the concealed tag in a cotton towel (top) and a woven fabric (bottom), (**c**) the tags visible under high light intensity.

The encapsulation process is completed by bonding the thermally molded Kapton onto the fabricated RFID filament using Masterbond EP37-3FLF adhesive and cured at 80 ${}^{\circ }$C for 3 h. Figure 4 shows the encapsulation steps of the RFID strip. Figure 5 shows the photographs of the encapsulated and textile-integrated tags.

## 4. Antenna Simulation and Measurements

**Figure 6:**
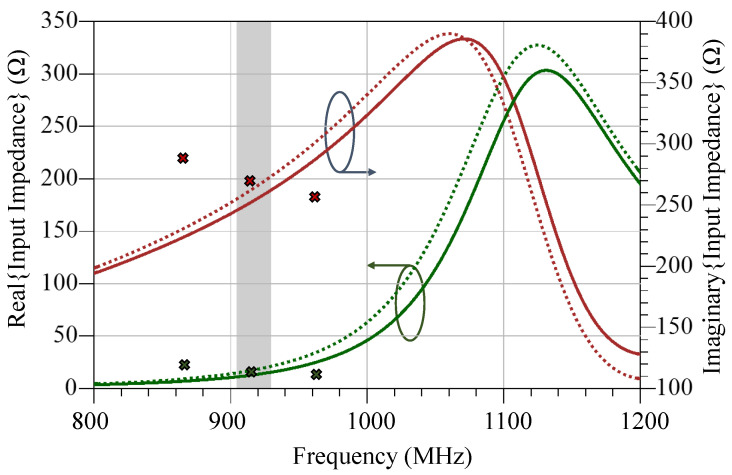
Simulated input impedance of the proposed antenna using Momentum MoM (dashed) and CST Finite Integration Technique (FIT) (solid) showing an impedance match, across the 915 MHz band, with the RFID IC’s impedance (shown as discrete points).

Full-wave Electromagnetic (EM) simulation has been used to compute the antenna’s input impedance and subsequently bandwidth. 2.5D Method-of-Moments (MoM) in Keysight ADS Momentum as well as 3D Finite Integration Technique (FIT) in CST Microwave Studio were used to simulate the antenna’s input impedance. The antenna has been modelled using a lossy copper model (Resistivity = 8 mΩ/square) on a Kapton polyimide substrate ($\epsilon_{r}$ = 3.2, tanδ = 0.02) at 915 MHz. The MoM solver has been used for initial optimization A lumped port has been used to provide a balanced excitation signal to the dipole antenna. Figure 6 shows the simulated antenna input impedance obtained from both MoM and FIT simulators. The observed discrepancy is in the magnitude of the antenna’s impedance response showing less than 5% higher impedance in the MoM simulator. This is attributed to the simplified approximation of the surface-roughness losses by the MoM as opposed to the 3D FIT CST solver. However, both simulators show an impedance match between the proposed antenna design and the RFID chip at the 915 MHz band.

The antenna’s reflection coefficient ($S_{11}$) can be calculated using the simulated and measured impedance. The power reflection coefficient and subsequently the read range of the tag can then be calculated using (1). The RFID tag’s $S_{11}$ is −21.1, −0.752, and −2.32 dB at 915, 866 and 953 MHz, respectively. Therefore, the antenna can be used with minimal power reflection between the IC and the antenna at 915 MHz (the design frequency).

The fabricated antenna’s impedance has been measured using a two-port Rohde & Schwarz ZVB4 Vector Network Analyzer (VNA) calibrated using a standard Through, Open, Short, Match (TOSM) calibration method. A broadband co-axial jig has been used to measure the differential impedance using the two imbalanced feeds from the VNA ports. The common and differential mode input impedances of the VNA are set to 50 Ω (the characteristic impedance of the VNA). The dipole impedance is then given by the differential impedance $Z_{d}$ (2).

(2) $$Z_{d}=\frac{2Z_{0}\left(1-S_{11}S_{22}+S_{12}S_{21}-S_{12}-S_{21}\right)}{\left(1-S_{11}\right)\left(1-S_{22}\right)-S_{12}S_{21}}.$$

For a symmetrical balanced antenna, the equivalent 2-port network is symmetrical and therefore (2) can be simplified to (3), where $S_{11}=S_{22}$, $S_{21}$ = $S_{12}$ and $Z_{0}$ = 50 Ω.

(3) $$Z_{d}=\frac{2Z_{0}\left(1-S_{11}^{2}+S_{21}^{2}-2S_{12}\right)}{{\left(1-S_{11}\right)}^{2}-S_{21}^{2}}.$$

**Figure 7:**
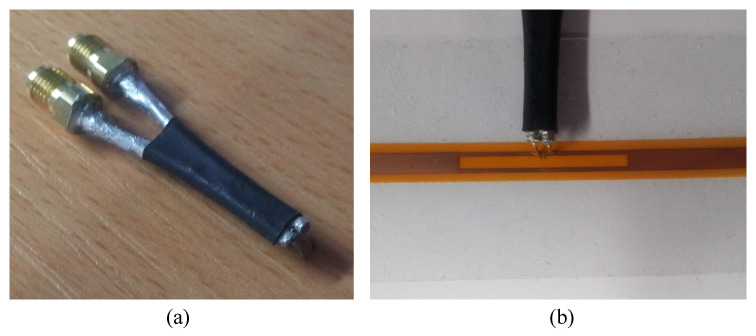
Photographs of the coaxial jig used to measure the antenna’s impedance (**a**) and the coaxial tips landing (**b**).

The antenna’s input impedance has been measured from 800 MHz to 1 GHz using the differential balanced test setup. The coaxial jig used to measure throughout impedance of the fabricated antenna is shown in Figure 7. The utilized method to measuring the input impedance of balanced dipole antennas has shown high accuracy and good agreement with numerically-simulated input impedances [33]. In addition, balanced ports have been previously used to measure the input impedance of textile-based [26] and flexible [34] RFID dipole antennas.

**Figure 8:**
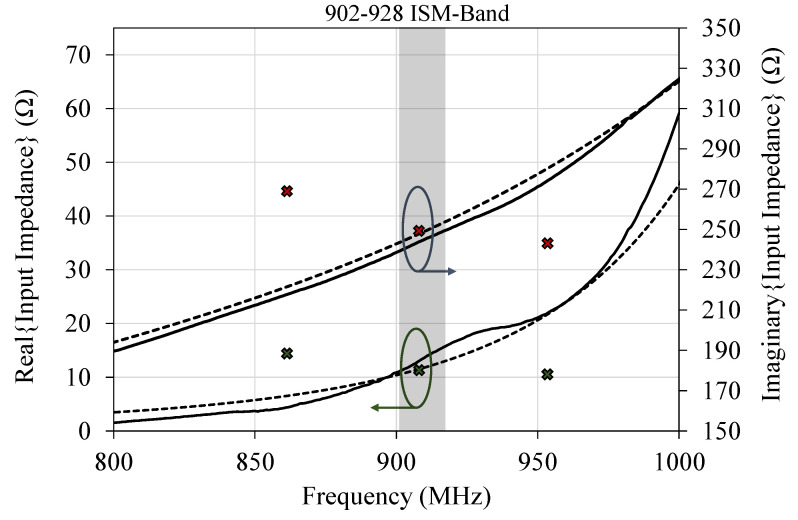
Simulated (CST: dotted line) and measured (solid line) input impedance (real: primary axis, imaginary: secondary axis) of the antenna showing a close match to the chip’s input impedance (shown as discrete points) at 915 MHz.

Figure 8 shows the measured and simulated input impedance of the antenna. The tag’s IC is shown for comparison as discrete points at 866, 915 and 953 MHZ, as reported in the datasheet. The tag’s IC is inclusive of the packaging effects and the soldering pads which provides a more accurate reference to evaluate the antenna’s impedance. Despite the imaginary IC impedance being shown on the positive imaginary impedance scale, it is noted that the antenna’s impedance is capacitive and the antenna is designed to achieve a complex conjugate match ($ℑ\left\{Z_{IC}\right\}=-ℑ\left\{Z_{Ant.}\right\}$).

**Figure 9:**
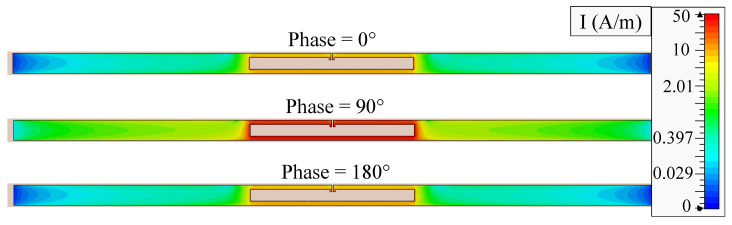
Simulated surface current magnitude plot of the antenna at 0${}^{\circ }$, 90${}^{\circ }$, and 180${}^{\circ }$ at 915 MHz.

**Figure 10:**
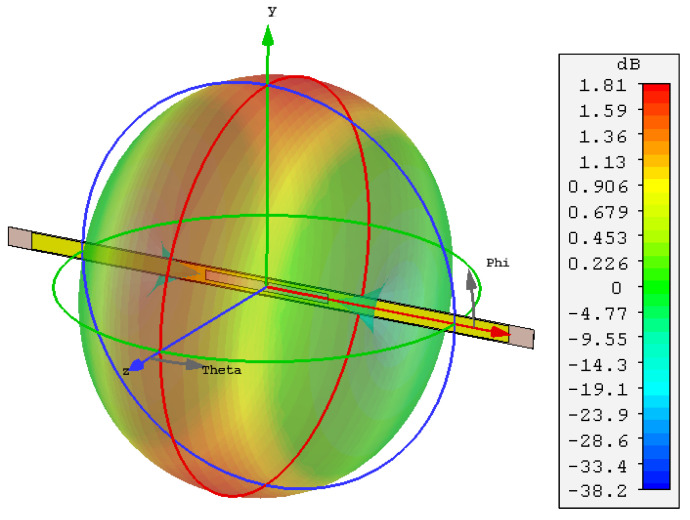
Simulated 3D far-field gain plot of the dipole antenna at 915 MHz, showing a peak gain of 1.81 dBi.

The antenna’s full-wave model in CST has been used to calculate the surface current of the antenna at 915 MHz. In Figure 9, it can be observed that the highest surface current distribution is seen at 90${}^{\circ }$ phase angle, showing a high surface current density across the entire dipole arms. The 3D far-field gain of the antenna, shown in Figure 10 shows the radiation patterns resembling a typical omnidirectional dipole antenna. However, the peak gain is less than the theoretical 2.1 dBi as the antenna is smaller than a half wavelength in length. To validate the simulated gain, analytical calculation of the radiation properties based on the surface current can be utilized.

A loop antenna’s directivity and gain can be calculated analytically based on the surface current integral [35], or simulated using numerical methods in a full-wave EM simulation software (CST Microwave Studio). While the proposed antenna is a folded-dipole antenna, the length *L* of the proposed antenna is used as the loop’s radius in the analytical gain calculations. The antenna’s directivity *D*, gain *G* are given by

(4) $$\begin{array}{c} G=\eta_{\mathrm{Rad}.}\times D \end{array}$$

(5) $$\begin{array}{c} D=\frac{120\pi^{2}{\left(ka\right)}^{2}}{R}\max\left\{J^{2}\left[ka\sin\left(\theta \right)\right]\right\}. \end{array}$$

**Figure 11:**
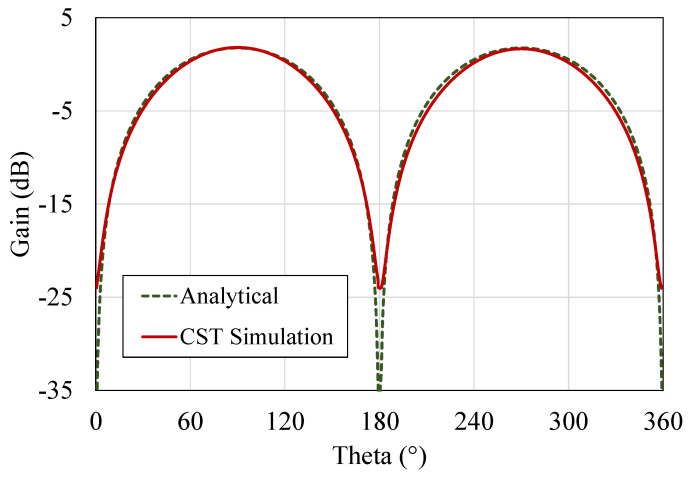
Analytically calculated and numerically simulated (CST) gain pattern of the antenna showing a close agreement and a 1.81 dBi gain.

The closed-form approximation in Reference [35] has been used to calculate the antenna’s *D*. Given the antenna’s size, and the high conductivity of the copper sheets, the radiation resistance of a half-wavelength dipole is over 100× the DC resistance. Thus, the antenna’s theoretical radiation efficiency approaches 100%. The analytically calculated antenna’s gain is shown in Figure 11 over the antennas E-plane.

The antenna’s gain obtained from the CST model is 1.81 dBi, which shows a very close agreement with the 1.76 dBi gain calculated analytically. Figure 11 shows the agreement between the analytical and the numerically-simulated gains of the RFID antenna. As the radiation patterns have not been measured experimentally, the agreement between the analytical calculation using (4) and (5) and the CST-simulated results validates the antenna’s gain patterns.

## 5. RFID Tag Testing and Evaluation

### 5.1. Tag Read-Range Test

The read range, *r*, of a RFID tag can be calculated using the Friis free space propagation loss model, and the RFID’s IC sensitivity. Using (1), the range of the antenna can be calculated using the 1.8 dBi gain (obtained from simulation and analytical calculations) and the |$S_{11}$| based on the impedance measurements, shown in Figure 8. The RFID reader in this work is a Zebra RFD8500 of 2.5 W (34 dBm) EIRP ($G_{t}P_{t}$) output, the incorporated chip’s −21 dBm sensitivity, and the 1.81 dBi RFID antenna gain are used to calculate the read range. The read-range has been calculated using a reflection coefficient of $S_{11}=-25$ dB at 915 MHz and $S_{11}=-1.5$ dB at 868 MHz, based on the IC’s impedance of 12.8−j248Ω.

Due to the transmission limits on the European RFID tag readers, confining the Zebra RFD8500 to 868 MHz, the read-range measurements were only performed for the 868 MHz band. Table 1 shows the measured and calculated read-range of the proposed RFID tag. It is seen that the calculated read-range of 10.4 m agrees within 21% with the measured 8.2 m read range, this represents the same error margin between the calculated and measured read-range in Reference [27], hence validating the calculated values and the simulation approach. The discrepancy can be attributed to additional DC losses due to the IC’s mounting process, resulting in a reduced impedance match and a lower radiation efficiency. In addition, the multi-path effects in an indoor test environment increase the propagation losses compared to an ideal free-space propagation model or an anechoic test range.

As the antenna aperture is a direct function of an electrically-small antenna’s size [36], both the tag’s read-range and the antenna’s read range need to be considered. To evaluate the performance of the proposed tag antenna, and compare it to commercially available UHF RFID tags and antennas reported in References [14,16,27,37], a Figure of Merit (FoM) is defined in (6) as a function of the read-range *R* in m normalized to the antenna’s area *A* in m${}^{2}$. This FoM is defined as it is directly linked to the antenna’s aperture efficiency. However, as the gain of RFID antennas is often not reported in literature, the read-range is compared instead of the gain as the main performance metric. Furthermore, UHF RFID antennas operate at the same frequency bands and hence the wavelength is not included in the FoM.

(6) $$\mathrm{FoM}=\frac{\mathrm{Read}\;\mathrm{Range}}{\text{Antenna’s}\;\mathrm{Area}}.$$

The performance of the proposed tag is compared to state-of-art RFID antennas and tags in Table 2. It can be observed that the proposed antenna achieves the highest FoM due to its compact size, while not compromising on the read range. Although a more complex RFID design such as the 3D printed cavity resonator in Reference [27] can achieve a higher read-range of up to 26 m, it occupies a significantly larger area and is non-planar, making it unsuitable for e-textile applications. Furthermore, the proposed thin polyimide filament allows seamless integration of the antenna in textiles without affecting the user’s comfort, where other textile antennas [14,16,37] utilize thick embroidered conductors or e-textiles which affect the fabric’s appearance and the user’s comfort. The method for weaving the antenna filmanet into the textile has been previously reported in References [2,28].

The read-range measurements and calculations have been performed in free space (i.e., in absence of a human body phantom). While human-proximity will affect the antenna’s impedance match, efficiency and gain, subsequently reducing the read-range, the main scope of this work is demonstrating a reliable textile-integrated RFID antenna using a compact antenna, and human interaction effects are not addressed. Studies [13,38], compared in Table 2, despite being implemented on flexible and wearable materials, have all been tested in free-space, using a similar approach to the test setup used in this work. On the other hand, where the reported antennas were tested on-body [12,14], we only compare the read-range values reported for free space operation for a fair comparison.

### 5.2. Practical Reliability Testing Tests

The designed RFID tag is proposed for wearable applications, as well as asset tracking of commercial textiles. Therefore, multiple standard tests (i.e., AATCC 135-2010, AATCC 150-2010 and AATCC/ASTM TS-006) were performed on the tag to evaluate its suitability for use in practical applications. Table 3 shows the test carried out to assess the tag’s reliability which is defined as the ability of the RFID tag to maintain its readability by a handheld reader for each test case.

The RFID filaments were integrated within a standard cotton towel and the reliability has been established by reading the RFID tag at a distance of 1 m during each test. These tests demonstrate the ability of the RFID tag to function in a number of real use case scenarios and were identified in conjunction with Invengo Technology SARL, the details of the washing test are in the next section. For example, the water immersion and wash tests demonstrate the waterproof of the filament assembly. The mechanical reliability of the tag and its ability to be read when attached to a metal case or when stacked with other RFID tags demonstrate the proposed encapsulated tag is suitable for retail and asset-tracking applications. Furthermore, the multi-tag read test demonstrates minimal mutual coupling and detuning when multiple antennas are placed in proximity.

### 5.3. Washing Test

The encapsulated RFID tags were tested against machine washing cycle to establish the maximum washing cycles that the encapsulation technique could undergo. The encapsulated RFID tags were manually sewn into the seam of a 15 × 15 cm${}^{2}$ face towel made of 100% cotton. Three towels were then loaded into a BEKO washing machine with detergent and softener as suggested by the manufacturer. The washing settings are included in Table 4.

A handheld RFID reader was used to read these tags during and after the washing to ensure the functionality. This test was repeated with the same three samples and the number of cycles when any of these samples stopped responding was noted down. The first reading failure appeared after 32 full washing cycles, the second failure appeared after 40 cycles and the third tag failed during the 50th washing cycle. Further investigation found that hairline cracks appeared on the areas between the antenna and RFID IC, causing water seeping though. It was also found that cracks happened on the copper layer close to the IC; it is believed that these cracks could be the results of an encapsulation failure at the same location.

## 6. Conclusions

In this paper, a textile-integrated RFID filament, that can withstand the harsh rigors of use, is presented. The RFID tag and antenna, fabricated on a thin flexible circuit filament achieve a 1.8 dBi gain and an impedance bandwidth covering the 915 MHz band. A read range of up to 8.2 m has been measured at 868 MHz with a maximum calculated range of 18 m at 915 MHz. The encapsulated RFID has been subjected to a series of rigorous reliability tests to demonstrate waterproofness, mechanical reliability, and the ability to be read at 1 m under various test conditions.

The proposed RFID tag is a demonstration of the feasibility of battery-free wireless communication to concealed e-textiles. Furthermore, this enables wearable textile-based battery-free RFID sensing embedded in clothing for future IoT/IoE applications such as healthcare monitoring, passive sensing, and asset tracking.

## Figures and Tables

**Table 1:** Calculated and measured indoor read-range of the proposed flexible RFID tag.

Frequency (MHz)	Calculated Range	Measured Range
868	10.4 m	8.2 m
915	18.0 m	-

**Table 2:** Comparison of the proposed RFID antenna with state-of-art RFID antennas.

Lit.	FoM (1/m)	*A* (mm)${}^{2}$	Max. *R* (m)	Thickness (mm)	Substrate	Flexible	Washable
Proposed	21,460	382	8.2	0.05	Polyimide	✓	✓
[27]	3095	8400	26	10	PLA cavity	✗	✗
[12]	680	6790	4.62	4	Textile	✓	✗
[14]	294	10,170	3	0.17	Textile	✓	✗
[13]	6840	1540	10.5	0.13	PDMS	✓	✓
[16]	3470	1876	6.5	-	Textile	✓	✓
[38]	2400	1650	3.96	1–2	Elastic polymer	✓	✗
[37]	2443	1023	2.5	1	Textile	✓	✗

**Table 3:** Test procedure of the textile-integrated RFID tag.

Test	Tag-Read	Details
Magnetic test	✓	A neodymium magnet is used to examine if the RIFD tag has Ferro-metals. This test checks whether the RIFD tag can be detected by the needle detector or MRI.
Metal strip test	✓	A copper foil is placed at back of RFID tag and a hand held reader is used to check whether the tag functions as expected. This test is for the food industry where the tag is used on metal cans.
Bending test	✓	A RFID tag is bent over a 2 cm diameter cylinder. The reader continuously reads starting from 0.5 m and is moved away until the tag is no longer readable.
Multi-stack test	✓	9 tags are randomly distributed in a box and the reader reads 9 tags at the same time.
Water Immersing test	✓	A RFID tag is submerged into water and read by a hand held reader, while in water.
Multi-stack test (wet)	✓	9 wet RFID cotton towels are stacked together and read by the reader.
Machine washing test and spinning	✓	1 cotton towel with a RFID tag is washed at 60 ${}^{\circ }$C for 40 min. The tag is spun at 500 rpm for 3 min. The tag is read before, during and after washing. This test examines tag under the most harsh washing condition specified by Invengo.
Drying test	✓	1 washed and spun cotton towel with a RFID tag is then dried at 180 ${}^{\circ }$C for 20 min. This test examines whether a tag can survive one drying cycle at the highest temperature specified by Invengo.

**Table 4:** Details of the washing procedure of the towel-integrated RFID.

Parameter	Setting
Washing temperature	60 ${}^{\circ }$C
Washing duration	40 min
Number of towels washed at the same time	3
Washing detergent	Daz regular washing pod
Softener	Fairy fabric softener original
Spin speed	600 RPM
Spin duration	6 min

## Datasets

Datasets presented in this paper will be made available from the University of Southampton repository at DOI:10.5258/SOTON/D1422.
